# A Quality Improvement Initiative to Transform Seasonal Immunization Processes Using Learning from the Coronavirus 2019 Pandemic

**DOI:** 10.1097/pq9.0000000000000716

**Published:** 2024-02-09

**Authors:** Eric D. Robinette, Pamela M. Nelly, Laurie J. Engler, Michael T. Bigham

**Affiliations:** From the * Department of Pediatrics, Akron Children’s Hospital, Akron, Ohio; † Department of Quality Services, Akron Children’s Hospital, Akron, Ohio.

## Abstract

**Background:**

Surge demands for annual influenza vaccines challenge healthcare systems. Mass immunizations differ from the traditional care model. The coronavirus 2019 (COVID-19) pandemic challenged current care models with amplified demand and infection risks while challenging the organization to create new and improve existing processes.

**Methods:**

Using the Model for Improvement, the team set out to (1) safely meet a surge in vaccination demand and (2) adopt pandemic-driven innovations into routine immunization practice.

**Results:**

This free-standing pediatric system delivered 87,000 COVID-19 vaccines (~1.3% state total). It administered over 50% of COVID-19 vaccines using new mass immunization processes, including 37,000 adult vaccines before pediatric authorization. In the 2021–2022 influenza season, it used the new or improved immunization processes to deliver 22% of influenza vaccines.

**Conclusions:**

Pandemic-driven adaptation for the COVID-19 vaccine substantially increased the efficiency of influenza vaccination processes but did not result in a clear increase in influenza vaccine administration rates.

## INTRODUCTION

### Problem Description

As the largest local provider of pediatric care in Northeast Ohio, Akron Children’s Hospital (ACH) faces an annual challenge of a surge in demand for influenza vaccinations. From September to November, the system gives over 40,000 doses of influenza vaccine, totaling 77% of the annual doses. Before the coronavirus 2019 (COVID-19) pandemic, the organization delivered most influenza vaccines using routine primary care processes, with some minor adaptations to seasonal demand. The organization anticipated that the successful development of COVID-19 vaccines would also create a massive demand for vaccinations, likely to strain its capacity. The existing annual influenza vaccine program offered an opportunity to test COVID-19 vaccination strategies under pandemic conditions.

### Available Knowledge

Many organizations use large-scale vaccine programs to meet seasonal or episodic demands for vaccination. The rapidly deployed COVID-19 immunizations created a large, time-sensitive demand^1^ and offered a logical extension of influenza vaccine work. COVID-19 created additional challenges beyond those faced with influenza. Most notably, the logistics of COVID-19 vaccines (eg, stringent cold storage requirements^2–5^) made distributing small quantities of vaccine to numerous sites difficult.^6^ This factor favored large-scale, single-site clinics. Historically, some organizations used large-scale, single-site clinics to meet sudden vaccine demand surges.^7,8^ Others have previously studied drive-through clinics and found them to be feasible,^7,9^ safe,^7^ satisfying for patients,^9^ efficient,^9^ and cost-effective.^10^ This also reduces the risk of droplet and aerosol transmission of COVID-19^11,12^ because drive-through patients remain in their cars. Thus, large-scale drive-through clinics offered a prime option for quickly increasing the organization’s influenza vaccine capacity during the COVID-19 pandemic.

### Rationale

**Fig. 1.**
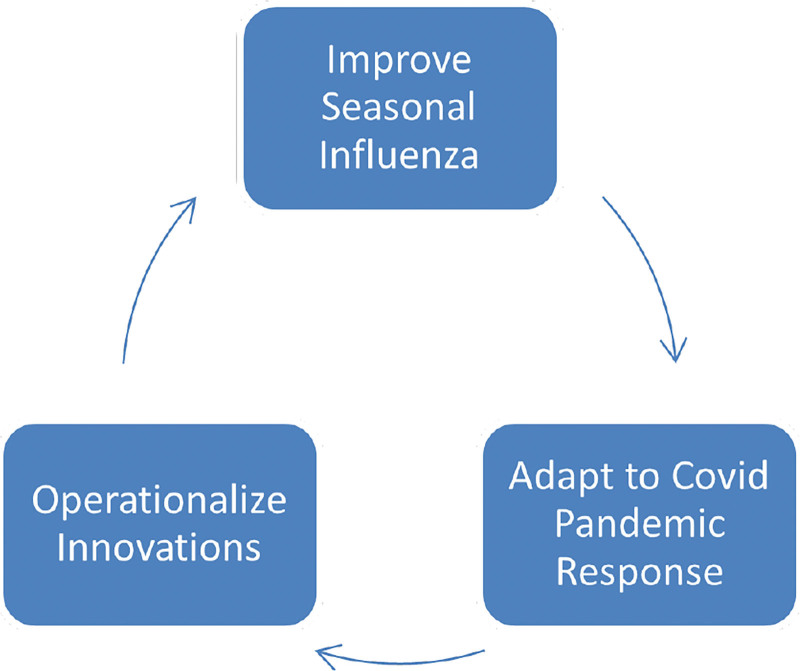
Testing model: testing with seasonal influenza, adapted with COVID-19, operationalize interventions, which influenced seasonal influenza vaccines.

We hypothesized that increasing the capacity to meet the surging demand for seasonal influenza vaccines using mass immunization clinics, particularly drive-through clinics, would increase overall influenza vaccination rates. We anticipated that this work would prove applicable to future COVID-19 immunization efforts. The resulting initiative would begin by leveraging an existing improvement team, processes, and tools developed for seasonal influenza. Influenza would serve as an initial testbed for new mass immunization processes before the availability of the COVID-19 vaccine. After that, continuous, iterative improvement of mass immunization processes and tools would flow freely back and forth between COVID-19 and influenza vaccines based on current demands (Fig. 1).

### Specific Aims

**Fig. 2.**
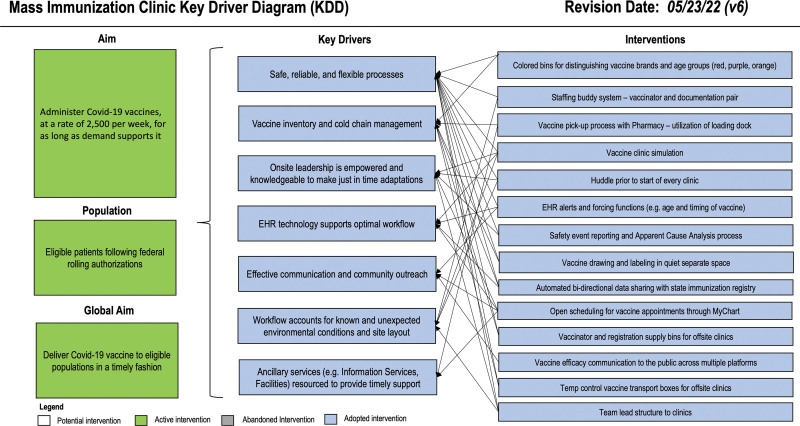
COVID-19 Immunization Clinic KDD. Our theory for COVID-19 vaccination clinics is modeled after learning from seasonal influenza vaccines.

For the influenza arm, the global aim was to increase influenza vaccine coverage in the ACH population to equal the most successful US states. The specific aim for influenza was to increase annual influenza vaccine administration rates from 48% to 60% by April 2022 (**See figure 1, Supplemental Digital Content 1**, which shows Influenza system-level key driver diagram (KDD). Our theory for improving influenza vaccine rates and mass immunization clinics. http://links.lww.com/PQ9/A540.) The global aim for the COVID-19 vaccine arm was to meet the anticipated surge in demand for COVID-19 vaccines after FDA authorization. ACH’s key populations for the COVID-19 vaccine included hospital employees and children. Local adult healthcare systems and public health departments were under tremendous volume-based stress when COVID-19 vaccines were first authorized for adults only,^13–16^ while the pediatric system was experiencing unprecedented low volumes. Thus, after the employee campaign, the improvement team expanded the target population to adults. They set a “best-guess” specific aim to administer 2,500 vaccines per week for as long as demand required (Fig. 2). However, this specific aim was difficult to define *a priori* due to the number of unknown variables, such as vaccine distribution schedules.

## METHODS

### Context

ACH is a pediatric-only, integrated health system serving Northeast Ohio. It comprises a 449-bed pediatric tertiary and quaternary care network with a flagship free-standing children’s hospital in Akron, Ohio. Additional free-standing and integrated pediatric and neonatal hospital locations, a full spectrum of sub-specialty services, and a 40+ office primary care network. The system sees over one million outpatient visits per year. ACH uses Epic (Epic Systems, Verona, Wis.), with a decade of EPIC experience, for clinical documentation and billing at all sites. Concerning vaccinations, at baseline, the electronic health record (EHR) was configured for routine operations and was not optimized for high patient throughput. Pre-pandemic ACH cared for a few adults. Thus, most adults seeking vaccines had neither an EHR record nor familiarity with the children’s hospital facilities.

The influenza vaccination initiative began in 2016 and was developed by 2019 into an institution-wide learning collaborative. The influenza vaccination initiative uses the Model for Improvement^17^ methodology. Two physicians, a QI specialist, and a project manager, comprise the initiative’s core leadership. Nineteen project teams of clinical staff and leaders from various clinical divisions within the hospital participate in the learning collaborative. During the run-up to the COVID-19 vaccination rollout, the organization asked the influenza vaccination initiative team to coordinate and lead the rollout of COVID-19 vaccinations. During major mass immunization activity for COVID-19, the core leadership team had access to human resources from across the organization to meet specific operational needs while continuing work with the original local teams from the influenza vaccination initiative.

### Interventions

The project core can be conceptualized as a large PDSA ramp to develop a safe, efficient, and flexible method for large-scale mass immunization clinics. The PDSA cycles flowed back and forth between influenza and COVID-19 vaccination initiatives based on seasonal demand for influenza vaccinations and the progressive rollout of COVID-19 vaccinations to various population subsets (Fig. 1). This flow unfolded in four overlapping phases:

1. Influenza Season One (2020–2021)
2. COVID-19 vaccine rollout (December 2020–December 2021)
3. COVID-19 vaccines adapting to routine care (June 2021–December 2021)
4. Influenza Season Two (2021–2022)

**Fig. 3.**
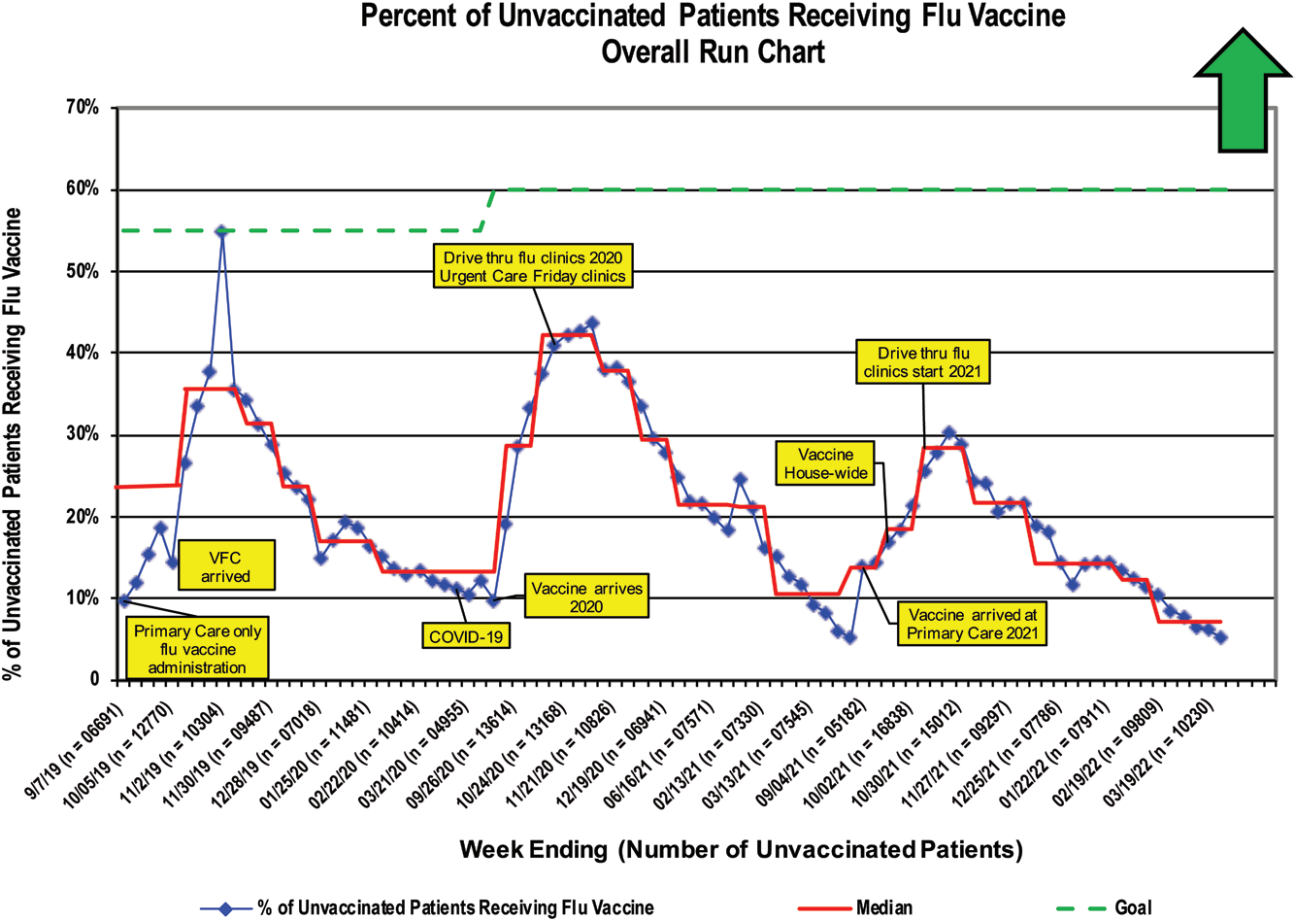
Influenza vaccine run chart, representing seasonal influenza vaccine administration rates throughout three seasons.

Notably, all four phases required adaptations to reduce the risk of COVID-19 transmission during the interventions. Supplemental figures 2 and 3 contain a chronological outline of the PDSA ramps and cycles and detailed explanations of the main processes/initiatives and potential failures. (**See figure 2, Supplemental Digital Content 2,** which shows Testing (PDSA) timeline for mass immunization clinics (influenza and COVID-19). http://links.lww.com/PQ9/A541.) (**See figure 3, Supplemental Digital Content 3,** which shows definitions and details around PDSA testing (influenza and COVID-19). http://links.lww.com/PQ9/A542.)

During the 2020–2021 influenza season, the team anticipated a future COVID-19 vaccine. Planning to test new interventions of mass immunization clinics for scale and safety under pandemic conditions started in the early summer of 2020. Simulations of drive-through mass immunization clinics followed the initial planning phase.^18^ The team then conducted an end-to-end test by vaccinating employees and testing all our processes except for the EHR. For example, traffic flow patterns were optimized, and a 20-minute postvaccination observation period was implemented by parking the cars in a supervised section of the lot. Finally, they held four 1-day, drive-through influenza vaccination clinics at four locations. The EHR component failed, resulting in subsequent PDSA ramps focused on integrating the EHR effectively. There were multiple points of failure, including network connectivity, the incompatibility of devices with outdoor use in adverse weather conditions, and an EHR documentation process that needed to be designed for high-throughput settings.

The COVID-19 vaccine rollout began in December 2020. Building on the experience with mass influenza clinics, ACH began with indoor mass COVID-19 immunization clinics for employees. Vaccines were documented directly into the employee health system, circumventing issues with the EHR. This approach allowed the team to focus on prototyping and iteratively refining the operational workflows and staffing. Subsequently, the mass immunization clinics expanded back to the drive-through and indoor off-site locations in the community for adult patients. Documenting into the state immunization registry rather than the EHR allowed a continued focus on optimizing clinical and operational workflows. At this point, new EHR workflows underwent prototyping and small-scale testing at indoor clinics. With the next stage of the COVID-19 vaccine rollout expanding to adolescents, the vaccine teams successfully scaled the prototype EHR workflows to large-volume clinics. Using a mobile EHR app also allowed the expansion of EHR use back to the outdoor drive-through clinics.

As COVID-19 vaccine supply improved and community demand declined, the focus shifted back to vaccinating in the primary care setting. The team piloted the new EHR workflow developed for the mass immunization clinics as the exclusive process for administering COVID-19 vaccines at two primary care sites. Feedback from this pilot resulted in adapting the new process for patients who required only the COVID-19 vaccine and continuing historical practices for those requiring both COVID-19 and other nonannual immunizations. The primary care network successfully scaled this adapted, hybrid process to six additional sites and then spread it to the 32 remaining sites before the next influenza season. The primary care network could subsequently accommodate the demand for the COVID-19 vaccine primary series and booster doses.

During influenza season two, the team spread the mass immunization clinic workflow, with full EHR and mobile device integration, to the drive-through influenza vaccine clinics. Specifically, processes for patient registration, vaccine ordering, and mobile app documentation piloted and then optimized through the COVID-19 mass immunization clinics made this possible. Several PDSAs were required to eliminate new issues with mobile devices, such as security access, logging into the system, workflow, and battery life. The primary care network adopted the EHR immunization clinic workflow previously piloted for COVID-19 vaccines for influenza and replaced old, cumbersome workflows. The improvement team then spread this workflow to sub-specialty offices.

### Study of the Interventions

This project used the objective outcome and process measures outlined below. The team relied on time-series analysis to establish a temporal connection between interventions and changes to the measures. The team also evaluated subjective information contemporaneous with the improvement activities. This included conducting formal debriefs, observing clinic operations, and working in various roles during some early clinics. They also collected and reviewed feedback from staff, standardized patients, and real patients.

### Measures

The primary outcome measure for influenza was the percentage of eligible patients presenting for an in-person visit who received an influenza vaccine at that visit. The measure defined eligible patients as those who met age requirements and did not have an influenza vaccine meeting the CDC-recommended vaccine schedule.^19^ The EHR determined vaccination status via the following sources: in-system vaccination records, historical immunizations manually entered during the visit, manual reconciliation of records obtained from the state immunization registry, and a limited set of external system records shared with our EHR. The team evaluated the total number of vaccines administered per week and clinic date as secondary process measures. For COVID-19, the project team used raw dose counts as an outcome measure. A denominator would have been difficult to determine or interpret due to the constantly changing eligibility criteria.^1,20–22^ During most key points in the COVID-19 vaccination campaign, community demand far exceeded the system’s supply, making system throughput an effective outcome measure. We did not have an *a priori* balancing measure. During the COVID-19 mass immunization clinics, we became concerned that the vaccine clinics were less accessible to some members of the community, resulting in racial inequity in vaccine access.^23^ We performed a post hoc analysis of our initial COVID-19 clinics to examine racial inequity relative to our region’s demographics.

Due to the seasonality of influenza and the rapidly changing nature of the COVID-19 pandemic, elements of context, such as persons eligible for COVID-19 vaccination and availability of influenza vaccine, varied throughout this work. Key contextual changes (eg, arrival of influenza vaccine) are annotated on the run charts. The project team used vaccination data from our internal EHR and the state immunization registry to validate key elements of the measures.

### Analysis

This project used run charts^17^ to track enterprise-level data for influenza and COVID-19 vaccinations and identify temporal associations between interventions and changes in the outcome measures. It also used small multiple-run charts at the local level to analyze the impact of local interventions. The team did not use formal methods to analyze qualitative data but extensively incorporated qualitative information into developing PDSAs and revising the KDDs.

### Ethical Considerations

This work was not human subjects research and thus not under the purview of the institutional review board. The team maintained a commitment to patient safety throughout this process, following procedures already in place at our institution and additional procedures specific to this work. These included simulation, mandatory staff training (eg, immunization administration and handling), standardized safety event reporting and review using the existing organizational process, extra safety accommodations for patients with medical and developmental issues affecting vaccine administration, contingency planning for issues unique to mass immunization clinics, and police presence for large clinics affecting use of public roads. Using established oversight processes at our organization, we adhered to CMS, OSHA, Ohio Department of Health, State Medical and Nursing Boards, and CDC guidance and regulations related to the scope of practice, worker safety, and vaccine handling and administration, including mandatory VAERS reporting. We monitored our programs for equitable access to vaccination.

## RESULTS

**Fig. 4.**
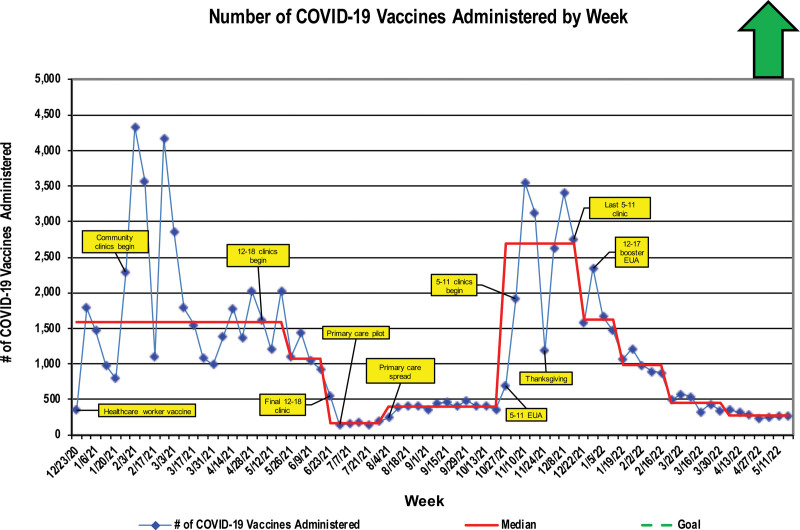
COVID-19 vaccination administration run chart. Peaks indicate the start of new mass immunization clinics and age group approval.

The full evolution of the interventions is detailed in Supplemental Figures 2 and 3. The data did not demonstrate a system-level increase in overall vaccination rates against influenza (Fig. 3). The mass influenza clinics in 2020 and 2021 delivered 832 and 1335 influenza vaccines, respectively. Four 1-day, single-site clinics constituted 1.17% (2020) and 1.7% (2021) of all the influenza vaccines administered by the organization. Mass immunization clinics yielded the vaccination of 45,727 staff, adult nonpatient community members, and children before July 2021. These clinics were the only mechanism for COVID-19 vaccination at this organization. This six-month total accounts for 52% of the total COVID-19 vaccines our organization gave between December 2020 and April 2022. Special cause degradation occurred on the run chart with the end of mass immunization clinics for 12- to 18-year-olds (Fig. 4). Special cause reflecting increased immunization rates occurred with rollout of the vaccine to the full primary care network in August 2021 and a second time in November 2021 (Fig. 4) with the launch of 5- to 11-year-old mass immunization clinics. After the end of mass immunization clinics for 5- to 11-year-olds, a steady trend of special cause degradation occurred. Ultimately, the entire COVID-19 vaccination program yielded 87,000 COVID-19 vaccines (~1.3% of the state total).

Concerning employee safety, no occupational cases of COVID-19 from working at a mass immunization clinic were identified via contact tracing. No serious patient safety events occurred at a mass immunization clinic. Finally, equity monitoring identified racial inequity in the population served by the early COVID-19 mass immunization clinics, and the team added additional sites in the community that successfully provided COVID-19 vaccine access to those underrepresented minorities.

## DISCUSSION

### Summary

This manuscript demonstrates a bi-directional, positive feedback improvement cycle. First, a preexisting seasonal influenza vaccination QI program created readiness for rapid improvement. This program then leveraged the early 2020–2021 influenza season as a testbed to develop a theory and process to inform an anticipated COVID-19 vaccination program. This knowledge jump-started the COVID-19 vaccination program, generating additional innovations through iterative improvement. The improvement team then fed these innovations from the COVID-19 vaccination program into subsequent influenza vaccination seasons, creating further improvement. Rigorous QI science formed the foundation for this virtuous improvement cycle.^17^

### Interpretation

The team adapted COVID-19 vaccination strategies from national experience^7,8,10^ and local, proactively designed PDSA cycles using influenza as a testbed. Community vaccination clinics,^7,8^ public-housing vaccination clinics,^24^ and corporate vaccine clinics^9,25^ before and during the COVID-19 pandemic have all been well-described in the literature. The unique features of our program are (1) the application of these strategies to adult patients under the leadership of a pediatric health system and (2) the real-time use of influenza vaccinations to prototype COVID-19 vaccines during an active pandemic. The flexibility of our pediatric health system enabled it to redirect its resources toward community vaccine delivery during a pandemic that largely spared children from hospitalization *en masse.* Thus, our pediatric system delivered 37,000 adult vaccines before pediatric authorization, assisting the local adult healthcare systems in focusing on managing their massive demand for care. For scale, 37,000 vaccines were roughly 60% of all the influenza vaccines our large (200+ provider) primary care network gave to its pediatric patients annually and a meaningful percentage of the total vaccines administered in our state. It is notable that despite the effectiveness of our mass immunization clinics, we only fully satisfied community demand once we fully integrated our primary care network into the processes. In most contexts, mass immunization clinics should be considered supplemental to efforts in the primary care setting, not a replacement for immunizations in primary care.

We did not anticipate the extent to which our learnings from COVID-19 would transform our approach to influenza vaccination. This constitutes a key learning from our work. Planned experiments to inform future adoption are foundational to the Model for Improvement.^17^ Our experience may be considered a combination of a planned and a natural experiment. While quite intentional, the innovations developed for COVID-19 were not necessarily to inform influenza vaccination but had a transformative impact on our processes. During the 2021–2022 influenza season, 22% of vaccines were delivered using new or improved immunization processes. The new workflows (1) save staff time during routine operations, (2) simplify the maintenance of our EHR by reducing variation and complexity, and (3) improve patient convenience in accessing influenza vaccines. It is also important to note that the “come to us” format of the mass immunization and drive-through clinics appeared to result in racial inequity. We had partial success compensating for this by adding “go to them” mass clinics at specifically targeted sites in the community where we were invited by community partners.

Despite these benefits, there was no absolute improvement in influenza vaccination rates. Several factors contributed to our inability to demonstrate improvement. First, there are substantial seasonal effects within the influenza vaccination rates data.^26^ The seasonal effects may obscure process and system improvements. Second, influenza vaccination rates have declined generally in our region after the COVID-19 pandemic.^27–29^ This secular trend may also confound the ability to interpret changes in our influenza vaccination rates.

### Limitations

The most important limitation in this improvement work is that although we achieved an increased capacity to deliver immunizations *en masse* within our organization, it needs to be clarified whether these patients would have accessed care through another organization. So, the effect on overall community vaccination rates still needs to be proven. Extraordinary resources were committed to fighting the pandemic. Most notably, the operational expenses of the COVID-19 mass immunization clinics were subsidized by the hospital, volunteers supplied some labor, and the government provided the vaccines at no charge. The COVID-19 pandemic applied atypical time pressures, which had positive and negative effects. Although this is a limitation for the applicability of this experience under normal conditions, it is also important to learn information relevant to future crises.

## CONCLUSIONS

A robust quality improvement (QI) capability and faithfulness to proven QI methods endows an organization with a degree of skill critical to the pandemic response. Although the circumstances of our project during the COVID-19 pandemic are unlikely to be repeated exactly, several timeless lessons emerge. The first is the importance of proactive, iterative testing. The second is that even when data quality is limited, collecting what data are available and feeding it into a theory-driven, rapid-cycle improvement process is still more effective than traditional problem-solving methods. A notable example from this work is how even unsophisticated QI analytics, like an un-denominated run chart, still provide meaningful feedback about the output of our complex system. Investing in organizational QI capacity is important for effective daily operations and crisis preparedness. During a crisis response like the COVID-19 pandemic, QI methods are not only feasible; they are essential.

## ACKNOWLEDGMENTS

The authors thank the following individuals for assistance with the study: Shana Earle, MBA; Jodi Simon, MSHA; Sarah Rush, MD; Elizabeth S. Smith, MPH; Brian Vandersall, MBA; Tori Wittmer, MSN, RN; Jessica Truesdell, MSN, RN, WCC; Mary Kay Walsh, BSN, RN; Kristin Wallace; Laura A. Chapman, PharmD; Christin Eland, MHA, BSN, RN; and the Akron Children’s Epic Ambulatory Analysts

## Supplementary Material
