# Supplementary material for: A Quality Improvement Initiative to Transform Seasonal Immunization Processes Using Learning from the Coronavirus 2019 Pandemic
Source: Pediatr Qual Saf. 2024 Feb 9;9(1):e716. doi: 10.1097/pq9.0000000000000716 (PMC10857672; doi:10.1097/pq9.0000000000000716)
Supplement: Supplementary file 1 [file pqs-9-e716-s001.pdf]

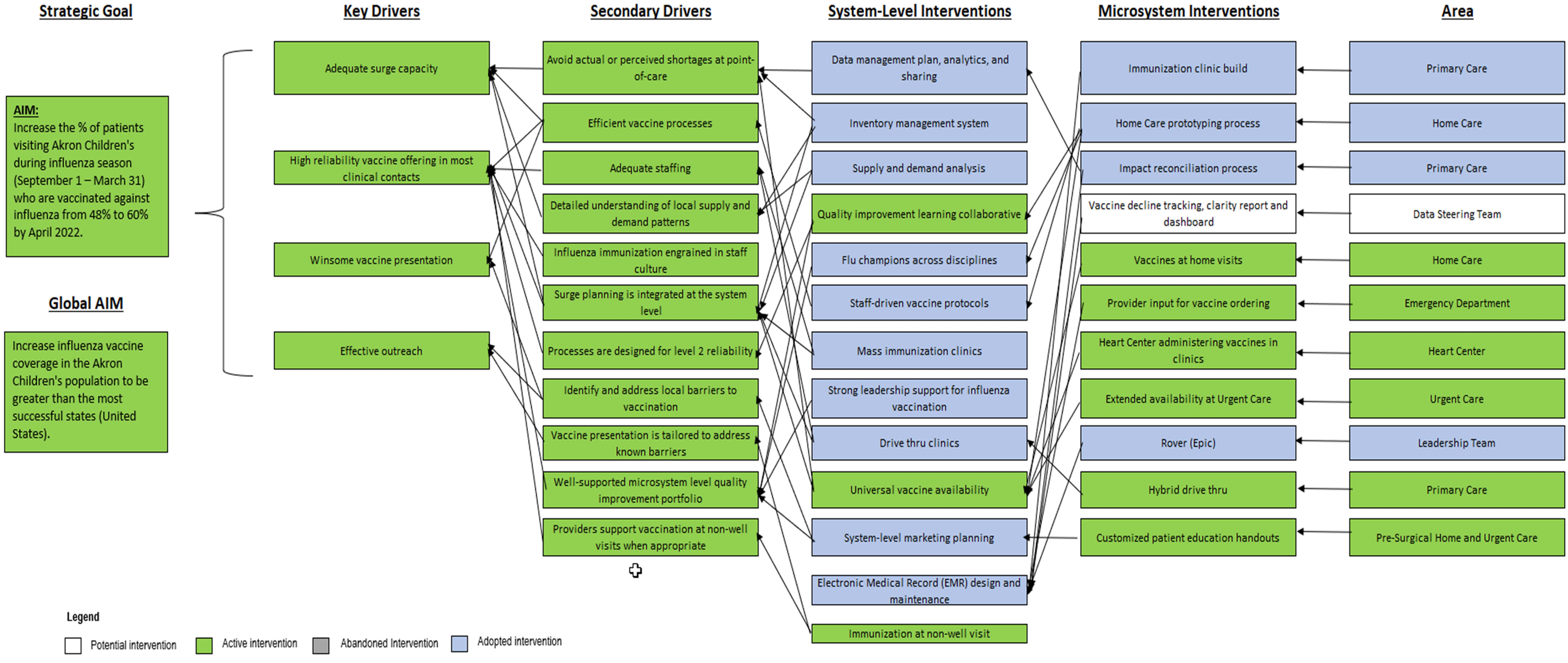
Strategic Goal
Key Drivers
Secondary Drivers
System-Level Interventions
Microsystem Interventions
Area
AIM:
Increase the % of patients visiting Akron Children's during influenza season (September 1 – March 31) who are vaccinated against influenza from 48% to 60% by April 2022.
Global AIM
Increase influenza vaccine coverage in the Akron Children's population to be greater than the most successful states (United States).
Adequate surge capacity
High reliability vaccine offering in most clinical contacts
Winsome vaccine presentation
Effective outreach
Avoid actual or perceived shortages at point-of-care
Efficient vaccine processes
Adequate staffing
Detailed understanding of local supply and demand patterns
Influenza immunization engrained in staff culture
Surge planning is integrated at the system level
Processes are designed for level 2 reliability
Identify and address local barriers to vaccination
Vaccine presentation is tailored to address known barriers
Well-supported microsystem level quality improvement portfolio
Providers support vaccination at non-well visits when appropriate
Data management plan, analytics, and sharing
Inventory management system
Supply and demand analysis
Quality improvement learning collaborative
Flu champions across disciplines
Staff-driven vaccine protocols
Mass immunization clinics
Strong leadership support for influenza vaccination
Drive thru clinics
Universal vaccine availability
System-level marketing planning
Electronic Medical Record (EMR) design and maintenance
Immunization at non-well visit
Immunization clinic build
Home Care prototyping process
Impact reconciliation process
Vaccine decline tracking, clarity report and dashboard
Vaccines at home visits
Provider input for vaccine ordering
Heart Center administering vaccines in clinics
Extended availability at Urgent Care
Rover (Epic)
Hybrid drive thru
Customized patient education handouts
Primary Care
Home Care
Primary Care
Data Steering Team
Home Care
Emergency Department
Heart Center
Urgent Care
Leadership Team
Primary Care
Pre-Surgical Home and Urgent Care
Legend
Potential intervention
Active intervention
Abandoned Intervention
Adopted intervention
