# Supplementary material for: A Quality Improvement Initiative to Transform Seasonal Immunization Processes Using Learning from the Coronavirus 2019 Pandemic
Source: Pediatr Qual Saf. 2024 Feb 9;9(1):e716. doi: 10.1097/pq9.0000000000000716 (PMC10857672; doi:10.1097/pq9.0000000000000716)
Supplement: Supplementary file 2 [file pqs-9-e716-s002.pdf]

Timeline Event	Date	PDSA Tested	Notes	Adapt/Adopt/Abandon
Influenza Simulation #1	9/9/20	Traffic flow and staffing assignments<br>Tested huddle script	Two-lane flow<br>Staff scripting<br>Update job descriptions	Adapted flow and huddle script
Influenza Simulation #2	9/12/20	Multi-car lane for vehicles with 3 or more passengers	Importance of walking physical space - flow changes from original diagram	Adapted two-lane flow and multi-car lane set-up
Influenza Simulation #3	9/18/20	Long drive-way and single entrance/exit, staff feared back-up	Tested process with stop-watch, unlikely that back-up would occur	Adopted physical space walk through
Influenza Simulation #4	9/19/20	Tested "runner" scenario	Helipad planning<br>Revised "runner" plans<br>Pull out section for vehicle break-down (had car with flat tire)	Adopted "runner" script into daily huddle<br>Adopted job descriptions<br>Adopted helipad plan and added to Beeghly huddle script
Employee Influenza Drive Thru Test	10/16/20	Tested buddy staffing (vaccinator and documentation pair)<br>Tested vaccine transport coolers	Staffing in pairs worked well<br>Two lane flow worked well, able to break down lane in afternoon	Adapt buddy staffing
Influenza Drive Thru Clinic #1	10/17/20	Scaled up buddy staffing<br>Tested lunch staffing plan	Families arrive early - be ready to start 30-minutes early<br>Need to have vaccine sitting on something while in cooler<br>Wi-Fi and EMR access issues - switched to paper<br>Importance of contingency planning	Adopted buddy staffing<br>Adopted two lane flow and multi-car lane process<br>Adapt lunch staffing
Influenza Drive Thru Clinic #2	10/22/20	Tested early start to accommodate early birds<br>Tested stacking vaccine on a basket while in cooler<br>Tested costumes and fun hats for staff to help distract kids during vaccine	Increase volumes<br>Include lunch details in staffing emails, no nearby food options<br>Order handwarmers - temps cold	Adopted start time for early birds<br>Adopted stacking vaccine in cooler on a basket<br>Adopted huddle script<br>Adapt costumes - need extras for staff who forgot to bring
Influenza Drive Thru Clinic #3	11/1/20	Tested cold weather supplies<br>Tested increased volumes	Cold temps, snow, rain and high winds had to use weather contingency plans and took down tents<br>Reduced to one lane flow in afternoon	Adopted cold weather supplies<br>Adapt clinic volumes - still have capacity
Influenza Drive Thru Clinic #4	11/8/20		Vaccine transport coolers would get too hot in direct unlight - PDSA solar covers at next drive thru clinic<br>Not a large turn out	
COVID-19 Vaccine Arrives	12/21/20	Tested clinic flow and drawing vaccine with 10 staff	Able to get the marketing photos and press portion completed before large-scale clinics started<br>Clinic flow worked well	Adopted three-room clinic flow, waiting area and check-out locations
Employee COVID-19 Clinics	12/22/20	Tested QR codes used for second dose appointments	Utilized MyLearning for appointment scheduling<br>Folks didn't always have phone or had older phone where QR code did not work	Adapt QR codes and adopted at later clinic with adjusted flow
Volunteer COVID-19 Clinic	1/16/21	Tested process for non-staff clinic flow	Employee clinic flow works well for community clinics	Adopt same clinic flow and staffing for community clinics
COVID-19 Drive Thru Clinic #1	1/29/21	Tested staffing process for an external organization	Communication challenges since not our event<br>Saw different documentation process in action	Abandon - Summit County Department of Health switched to hiring contract nurses after three clinics
Community COVID-19 Clinic #1	2/8/21	Tested Immunization Clinic build<br>Tested Epic Registration	Access challenges<br>Immunization Clinic build worked great	Adopt Immunization Clinic build and spread<br>Adapt access and education process
Community COVID-19 Clinic #2	3/3/21	Tested new screening process<br>Tested vaccine stations with signage<br>Tested to go supply boxes	Four step process tested: registration, screening, vaccine, wait - process worked well, and reading screening questions helped	Adapted new screening process for offsite clinics<br>Adapted vaccine stations for offsite clinics<br>Adapted supply boxes
Housing COVID-19 Clinic #1	3/10/21	Tested color-coding system for supply boxes	Registration confusion - checking-in folks followed by our team<br>Location challenges: construction, parking, small space to vaccinate	Adopted new screening process for offsite clinics<br>Adopted vaccine stations for offsite clinics<br>Adapted supply box PDSA
Corporate COVID-19 Clinic #1	5/11/21	Tested par levels and checklists to supply boxes	Corporations took care of scheduling - experienced increased numbers of no shows	Adopted supply box, color-coding, par levels and checklists
COVID-19 Drive Thru Clinic #2	6/5/21	Tested solar covers for vaccine coolers, when in direct sun<br>Rover test - EMR registration and documentation	Second clinic where turn-out was minimal - alternative location in the future	Adopted costumes and color dress themes for vaccine clinics<br>Adapt solar covers<br>Abandon Mansfield as drive thru location<br>Adapt Rover process
COVID-19 Drive Thru Clinic #3	6/12/21	Tested as drive thru location<br>Continued solar cover test<br>Rover test continued	Great turn-out and parking lot has more than enough space for a drive thru clinic	Adopted location for future drive thru clinics<br>Adopted solar covers<br>Adapt Rover - login issues
Influenza Drive Thru Clinic #5	10/3/21	Tested having both flu and Covid vaccines available<br>Rover test	Increased Urgent Care volumes - parking challenge for 15-minute wait<br>Moved to paper due to Rover access issues	Adopted multiple vaccine available<br>Adapt Rover
Influenza Drive Thru Clinic #6	10/10/21	Rover test	Had some documentation issues with Rover that were resolved quickly - started day on paper then moved back to Rover	Adapt Rover
Influenza Drive Thru Clinic #7	10/17/21	Rover test	Access issues resolved - Rover worked well!	Adapt Rover
Influenza Drive Thru Clinic #8	10/24/21	Tested as drive thru location<br>Rover test	Great turn-out and support from community<br>Rainy weather<br>Rover continued to work well - even in the rain	Adopted location<br>Adopt Rover
