# Supplementary material for: A Quality Improvement Initiative to Transform Seasonal Immunization Processes Using Learning from the Coronavirus 2019 Pandemic
Source: Pediatr Qual Saf. 2024 Feb 9;9(1):e716. doi: 10.1097/pq9.0000000000000716 (PMC10857672; doi:10.1097/pq9.0000000000000716)
Supplement: Supplementary file 3 [file pqs-9-e716-s003.pdf]

Process Term or Workflow	Definition
Huddle Script	Team discussion (15-min) at start of every clinic with expected day events, times, number of patients, potential problems/actions, introduction of team members and roles, flow of traffic (patients' indoor clinic and cars if drive-through).
Staffing Assignments	Staffing was determined by the following roles: registration (indoor/outdoor), vaccinator, documenter, and traffic control.
Buddy Staffing	Buddy staffing was used for the vaccinator /documentation– Pairing two staff members; one who documented and one who administered the vaccine within the Epic system.
Lunch Staffing Plan	went through several PDSA cycles including adjusting patient scheduling, rotating staff, staff restriction from leaving clinic site due to time constraints and lack of available restaurants to drive-through sites.
Job Description	roles and responsibilities of duties to be performed at clinic was communicated to staff via email prior to clinic dates.
Costumes	Seasonal costumes, hats, shirts used to entertain and distract children and reduce patient anxiety.
Traffic Flow	Determined by location, size, and orientation of clinic (indoor vs outdoor drive-through).
Two Lane Flow	based upon car occupancy- first lane= single car w/1-2 patients – second lane = >2 patients.
Rover Test	Cell phones with an Epic application, Rover, designed to register, screen, document vaccine administration in real time. Registration within the Rover application did not work efficiently and was abandoned.
Runner Scenario	Simulation was used to identify potential failures such as a child running from the family's car into potential traffic and other possible events such as a car breakdown, emergency event, and med-flight helicopter arrival during the drive-through clinic with a helipad nearby.
Supply Box	Plastic boxes with lids used to transport supplies with specific workstation needs which were addressed with a check list attached to the lid with par levels. These were replenished at the conclusion of each clinic and filled according to the par levels for the next clinic prior to the day of the clinic.
Color Coded Boxes and Labels	specifically used during Covid to add a layer of safety identifying appropriate vaccine dosage for matching patient age ranges (ie. red, purple, orange, gray, vial label matched box color and vaccine cap/label).
Vaccine Transport Box	Cooling boxes specifically designed for the transportation of vaccines. They contain temperature tracking devices to ensure that vaccines maintained optimal temperature control and the data can be downloaded for regulatory purposes. Solar covers/blankets were used to protect the boxes from direct sunlight and heat during warm temperatures.
